# Ayurvedic Phytochemicals in Oncology: ADP-Ribosylation as a Molecular Nexus

**DOI:** 10.3390/cells14221753

**Published:** 2025-11-10

**Authors:** Gali Sri Venkata Sai Rishma Reddy, Suman Kumar Nandy, Pitchaiah Cherukuri, Krishna Samanta, Pulak Kar

**Affiliations:** 1 Department of Biological Sciences, SRM University-AP, Amaravati 522240, India; sairishmareddy_gali@srmap.edu.in (G.S.V.S.R.R.); pitchaiah.c@srmap.edu.in (P.C.); 2 BIRAC’s BioNEST Bio-Incubator (B3I) Facility, North-Eastern Hill University, Tura Campus, Tura 794002, India; bhramoman@gmail.com; 3 Department of Biotechnology, Koneru Lakshmaiah Education Foundation, Vaddeswaram, Guntur 522302, India

**Keywords:** cancer, ayurvedic phytochemicals, ADP-ribosylation (ADPr), poly (ADP-ribose) polymerase (PARP), apoptosis, Phyto-nanomedicine, translational therapeutics, immunomodulation

## Abstract

Cancer remains one of the most pressing health challenges of the 21st century, with rising global incidence underscoring the need for innovative therapeutic strategies. Despite significant advances in biotechnology, curative outcomes remain limited, prompting interest in integrative approaches. Ayurveda, the traditional Indian system of medicine, suggests a holistic therapeutic framework that is now gaining molecular validation in oncology. In this review, the literature was systematically collected and analyzed from major databases, including PubMed, Scopus, and Web of Science, encompassing studies across ethnopharmacology, biochemistry, and cancer biology. The analysis focused on Ayurvedic phytochemicals that modulate ADP-ribosylation (ADPr), a dynamic post-translational modification central to DNA repair, chromatin organization, and cellular stress responses, with particular emphasis on poly (ADP-ribose) polymerase (PARP)-mediated pathways and their oncological relevance. We have also explored the role of p53, a key stress-response regulator intricately linked to ADPr dynamics, which acts as a downstream effector integrating these molecular events with cell fate decisions. Evidence indicates that several Ayurvedic compounds, including curcumin, resveratrol, and withaferin A, influence PARP–p53 signaling networks, thereby modulating DNA repair fidelity, apoptosis, and tumor adaptation. The review further addresses challenges related to the poor solubility of these phytochemicals and highlights recent advances in Phyto-nanomedicine-based delivery systems that enhance their stability and therapeutic efficacy. Overall, the synthesis of Ayurvedic pharmacology with molecular oncology reveals mechanistic insights that may inform the rational development of novel, mechanism-driven cancer therapeutics.

## 1. Introduction

Cancer arises through a gradual accumulation of genetic and epigenetic alterations that disrupt normal cellular control, leading to malignant transformation and tumour heterogeneity across different cancer types [1,2]. Landmark discoveries such as oncogene activation—through viral mechanisms like the Rous sarcoma virus or via mutations in proto-oncogenes, have laid the foundation of modern cancer biology [3]. Although both viral and non-viral origins of cancer are well recognized, a unified mechanistic understanding of tumour progression remains elusive. Recent studies highlight the interplay among driver mutations, epigenetic regulators, signalling proteins, and metabolic pathways, alongside the critical influence of the tumour microenvironment (TME), immune system, and microbiome in shaping cancer initiation and therapy response [4]. Within this context, tumour metabolism, particularly involving the TME and infiltrating immune cells, underscores the dynamic adaptability of cancer and its resistance to therapy [4,5]. While complete prevention may be unrealistic, early detection remains key to improving survival outcomes [5]. Despite advances in targeted and immune-based therapies, challenges such as toxicity, financial burden, and reduced quality of life, especially from oral oncolytics—persist [6,7,8].

In response, there has been a growing global shift toward integrative cancer care that emphasizes early screening, preventive measures, supportive therapies, and palliative management. Complementary and alternative medicine (CAM) has gained increasing attention for its ability to enhance patient well-being and reduce treatment-associated complications [9,10]. However, treatment responses are often accompanied by challenges such as therapy resistance, tumour recurrence, and cancer stem cell regeneration, along with potential off-target effects on intracellular organelles, all of which collectively limit therapeutic efficacy [10,11]. Among the various CAM modalities, Ayurveda, the ancient Indian medical system, has emerged as a promising adjunctive approach, particularly for its role in strengthening immunity and supporting post-treatment recovery [4]. With a history of more than 5000 years and foundations in classical texts such as *Charaka Samhita*, *Sushruta Samhita*, and *Ashtanga Hridaya*, Ayurveda offers a holistic framework for health and disease management [4]. Contemporary studies have reported its potential to alleviate symptoms and improve the quality of life in cancer patients [12,13,14]. Nevertheless, issues such as lack of standardization, limited reproducibility, and inadequate clinical validation continue to hinder its broader integration into global oncology frameworks [15].

In recent years, advances in nanoscience and molecular medicine have opened new avenues to modernize Ayurvedic formulations, particularly through green nanotechnology, offering a scientific bridge between traditional wisdom and contemporary biomedical innovation [16,17]. For example, Nano Swarna Bhasma (NSB), a gold nanoparticle formulation developed through green nanotechnology, has shown promise in metastatic breast cancer by enabling targeted delivery of bioactive phytochemicals such as γ-terpinene, α-pinene, cuminaldehyde, quercetin, kaempferol, and apigenin [15,18,19]. These strategies improve bioavailability and reduce systemic toxicity, creating a foundation for more standardized and reproducible therapeutic platforms [15,18,20,21,22]. Ayurvedic cancer interventions include polyherbal formulations, dietary modifications, detoxification procedures, and lifestyle practices aimed at reducing inflammation and oxidative stress while strengthening immune function [4,15,23,24].

Earlier Ayurvedic studies often focused on single compounds like curcumin or withanolides, but the multifactorial nature of cancer demands multi-targeted approaches [25]. Ayurveda’s holistic perspective may provide an advantage by influencing complex cellular pathways such as ADPr [26,27]. This post-translational modification regulates DNA repair, chromatin remodelling, inflammation, and apoptosis, making it highly relevant to cancer progression and therapy resistance [26,27]. Although direct evidence connecting Ayurvedic formulations to ADPr is limited, herbs such as *Withania somnifera* and *Curcuma longa*, known for their antioxidant and anti-inflammatory properties [28,29], may affect upstream processes like oxidative stress and DNA damage response that converge with ADPr signaling [30,31]. *Rasayana* therapies, traditionally used to promote vitality and immune resilience, may also influence these mechanisms, offering a promising yet underexplored direction for integrative oncology [32]. Despite centuries of empirical use, strong clinical validation remains limited [4]. This review consolidates current evidence on the molecular basis of Ayurvedic interventions, highlighting their effects on inflammation, immune modulation, oxidative stress, tumor signaling, and microenvironment [15,18,20,21,22], and explores their potential role in biomarker-driven cancer diagnostics and therapeutics. It presents a comprehensive analysis of the emerging interplay between Ayurvedic phytomedicine and cellular ADPr, a post-translational modification mediated by PARPs, which are tightly coupled to the DNA damage response and implicated in various diseases, including cancer. By bridging traditional medicine with molecular oncology, this work underscores the untapped potential of Ayurvedic principles in elucidating cancer mechanisms, advancing preventive strategies, and shaping next-generation therapeutic paradigms. Ultimately, it envisions a future where ancient wisdom and molecular precision converge to redefine the model of personalized cancer care.

## 2. Selected Ayurvedic Phytochemicals in Cancer-Mechanistic Insights into Molecular Targets and ADP-Ribosylation

**Figure 1:**
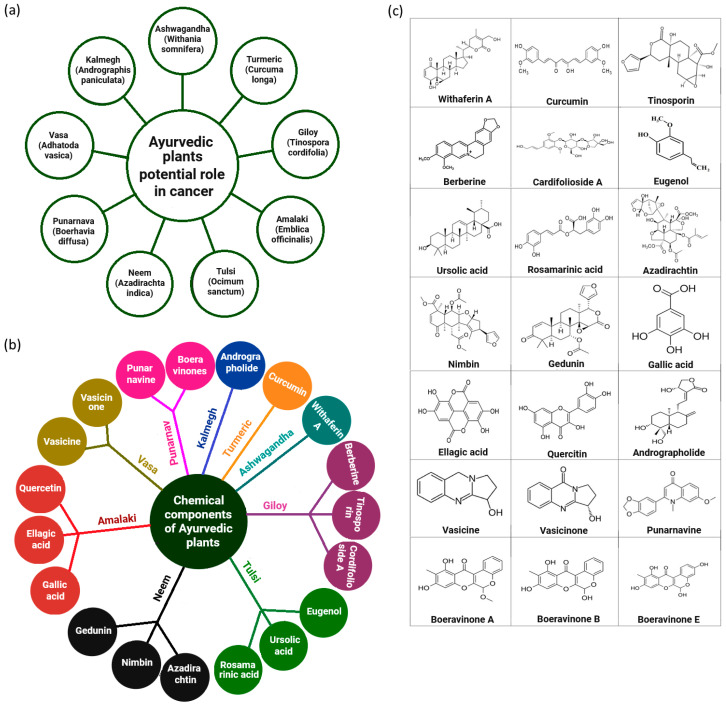
Ayurvedic phytochemicals exhibit promising potential in cancer therapy, with numerous compounds already recognized for their significant anticancer properties. (**a**) Highlights key Ayurvedic plants with potential roles in cancer management, while (**b**) depicts the major bioactive phytochemicals derived from these plants. (**c**) Presents the chemical structures of selected anticancer phytochemicals of Ayurvedic origin. For detailed insights into their molecular mechanisms, including involvement in ADPr and associated crosstalk pathways, please refer to the main text and corresponding bibliographic references.

Ayurvedic plants and their bioactive constituents modulate key signaling pathways involved in cancer progression, including inflammation, apoptosis, cell cycle regulation, and angiogenesis. Elucidating their molecular targets offers valuable insights into their therapeutic potential in cancer management [33]. Several compounds illustrated in Figure 1 exhibit anticancer effects through direct interactions with specific molecular targets. While the established activities of Ayurvedic phytochemicals largely stem from their ability to regulate oxidative stress, inflammation, apoptotic signaling, and cell cycle checkpoints, emerging evidence suggests their possible involvement in ADPr signaling, particularly within the DNA damage response and programmed cell death pathways. ADPr, catalyzed by enzymes such as PARPs and mono (ADP-ribosyl) transferases, represents a crucial post-translational modification that governs DNA damage sensing and repair, chromatin remodeling, transcriptional regulation, and cell death mechanisms including apoptosis, necroptosis, and parthanatos [34,35].

Although the present study does not include extensive experimental validation directly linking PARP mediated ADPr with specific phytochemicals, the conceptual framework has been strengthened by integrating recent computational insights. Several investigations have demonstrated the mechanistic feasibility of phytochemical interactions with PARP enzymes through molecular docking and simulation-based analyses. A recent study applied 3D QSAR, docking, and molecular dynamics simulations to curcumin analogues, revealing strong binding affinity and modulation of PARP1 activity [36]. Another work employed a multistage virtual screening approach combining 3D pharmacophore modelling, docking, and molecular dynamics simulations to identify selective PARP1 inhibitors [37]. The development of CDK1 PARP1 dual inhibitors further highlights the translational relevance of Ayurvedic phytochemicals as promising anticancer scaffolds [38]. Computational studies have also examined PARP7 modulation and its association with androgen receptor signalling in prostate cancer [39,40]. Moreover, recent evidence suggests that machine learning approaches can enhance virtual screening accuracy for PARP1 inhibitors [41]. These studies provide molecular level evidence supporting phytochemical PARP ADP ribosylation crosstalk and underline the need for experimental validation within the Ayurvedic phytochemical context.

**Figure 2:**
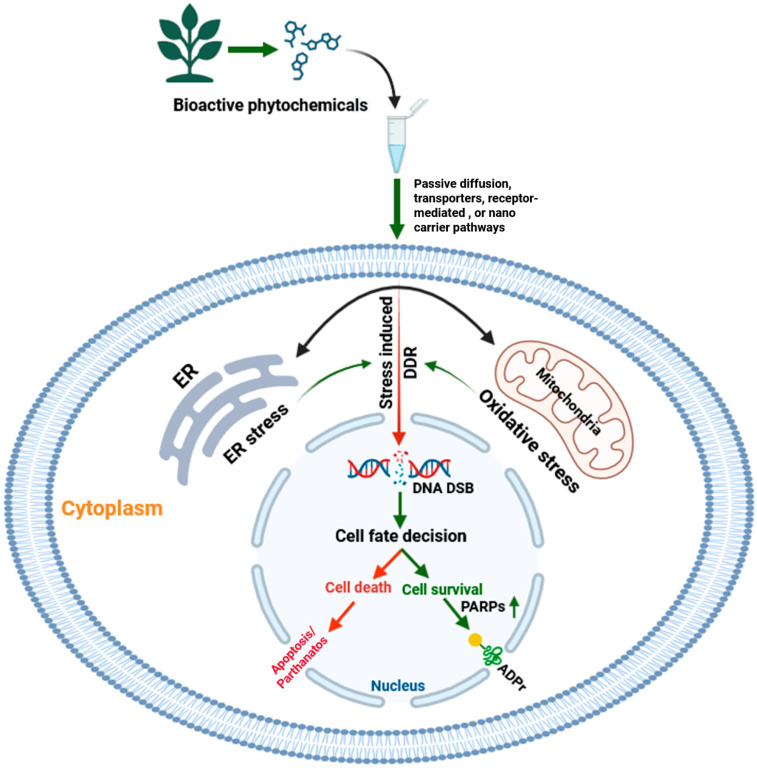
Proposed model of phytochemical-mediated modulation of cellular stress and ADP-ribosylation signaling. Bioactive phytochemicals from medicinal plants may enter cells via passive diffusion, transporters, or receptor-mediated pathways, influencing endoplasmic reticulum (ER) and mitochondrial stress responses. These stresses activate the DNA damage response (DDR), particularly double-strand breaks (DSBs), leading to PARP1-driven ADPr (green arrow). Moderate activation promotes DNA repair and survival, whereas excessive activation depletes NAD^+^ and triggers cell death, apoptosis, or parthanatos (red arrow). The model proposes that phytochemicals fine-tune this balance, sensitizing cancer cells to apoptosis or enhancing repair, offering new directions for DDR-targeted therapies.

Direct experimental evidence linking Ayurvedic compounds to the modulation of PARPs or other ADP ribosyl transferases remains limited; however, their indirect cellular actions such as triggering DNA damage, influencing NAD^+^ metabolism, and promoting oxidative stress suggest a potential regulatory impact on ADPr signaling pathways. As illustrated in Figure 2, bioactive phytochemicals may enter cells through passive diffusion, transporter-mediated uptake, or receptor-triggered signaling. Once internalized, they can influence endoplasmic reticulum and mitochondrial oxidative stress, leading to activation of the DNA damage response (DDR), particularly DNA double strand breaks. In this context, PARP1 becomes activated and catalyzes ADPR to facilitate DNA repair and promote cell survival. However, excessive or prolonged activation can deplete cellular NAD^+^ and trigger programmed cell death.

This conceptual model suggests that Ayurvedic phytochemicals may fine-tune these processes, sensitizing damaged cells toward apoptosis or enhancing repair and survival mechanisms depending on the stress level. Such modulation of DDR and ADPr pathways could have significant therapeutic implications, especially when combined with PARP inhibitors in DNA repair deficient cancers such as BRCA mutated breast and ovarian tumors [42,43]. Further experimental validation of these interactions may provide new directions for integrating Ayurvedic phytochemicals into modern oncotherapy. This underexplored mechanistic interface underscores the potential of Ayurvedic phytochemicals as adjunctive agents in PARP-targeted therapies. The subsequent sections therefore provide an overview of key Ayurvedic plants, their active phytoconstituents, and the molecular mechanisms underlying their anticancer effects, with emphasis on emerging links to genome stability, oxidative stress regulation, and ADPr signaling pathways.

Quercetin (3,3′,4′,5,7-pentahydroxyflavone): Quercetin is a naturally occurring polyphenolic flavanol abundantly found in onions, grapes, berries, cherries, broccoli, and citrus fruits [44]. It is also widely distributed across plant species such as *Santalum album*, *Mangifera indica*, *Emblica officinalis*, *Curcuma domestica*, *Withania somnifera*, *Foeniculum vulgare*, and *Cuscuta reflexa* [45]. Exhibiting antioxidants, anti-inflammatory, antifungal, antiviral, anti-allergic, and anticancer activities, quercetin protects against oxidative stress, tissue injury, and drug-induced toxicities [44]. Mechanistically, by inhibiting PARP-mediated single-strand break repair, quercetin increases unrepaired lesions in homologous recombination-defective (BRCA2-deficient) cells, leading to double-strand break accumulation and selective cytotoxicity, supporting its role as a small-molecule modulator of ADPr-linked DNA repair pathways [46]. It broadly modulates oncogenic signalling by inhibiting PI3K/AKT/mTOR and MAPK/ERK pathways, elevating Bax/Bcl-2 ratios, activating caspase-3/-9 and cytochrome c release, and reducing matrix metalloproteinase expression [47]. Although direct evidence linking quercetin to PARP family proteins remains limited, its induction of DNA damage and redox perturbation likely enhances PARP/ADPr activity, while inhibition of survival signalling may impair repair efficiency. From an Ayurvedic and nanomedicine standpoint, its poor bioavailability has led to nano-formulation strategies offering translational potential in ADPr-modulated oncology. Moreover, quercetin modulates ADPr signalling through Sirtuin 6 (SIRT6), a nuclear NAD^+^-dependent deacetylase and mono-ADP-ribosyl transferase that regulates DNA repair and chromatin remodeling via ADPr of PARP1 (Lys521) and BAF170 (Lys312), thereby promoting double-strand break repair and NRF2-dependent transcription [48,49]. Quercetin regulates SIRT6 activity in a dose dependent manner, acting as an inhibitor at low concentrations and an activator at higher doses, as supported by mass spectrometry, molecular docking, and molecular dynamics analyses [49,50]. This dual modulatory behaviour underscores its potential to fine-tune ADPr signalling controlling genomic stability, metabolism, and tumour suppression. Although direct experimental evidence of quercetin-mediated PARP modulation remains scarce, future studies dissecting its influence on PARP/SIRT crosstalk in the DNA damage response may reveal crucial insights into its anticancer potential.

Rosmarinic acid (RA) [*Rosmarinus officinalis* L. (Lamiaceae)] is a natural polyphenolic ester of caffeic acid and 3,4-dihydroxyphenyllactic acid, first isolated from *Rosmarinus officinalis* (rosemary) [51,52]. Abundant in Lamiaceae herbs like rosemary, lemon balm, mint, and sage, RA exhibits antioxidant, anti-inflammatory, antimicrobial, and anticancer activities [51]. Its anticancer effects are linked to suppression of neoplastic transformation and bone metastasis in breast cancer via downregulation of receptor activator of NF-kappa B ligand (RANKL) and interleukin-8 (IL-8) [51,53], reversal of multidrug resistance by targeting multidrug resistance by downregulating MDR1 and P-glycoprotein (P-gp) in gastric cancer [51], and modulation of MAPK/ERK and ERK/PKA pathways in several cancer models [51,53]. Notably, Su et al. (2017) reported weak but measurable inhibition of PARP-1 (~20% at 100 µM) by RA, suggesting a potential role in ADPr-linked DNA repair, especially under BRCA2-deficient conditions [54]. Further investigation into RA’s impact on cellular ADPr may support its development as an anticancer agent. However, its clinical use remains limited due to poor bioavailability, which may be improved through nanotechnology-based delivery strategies [55].

Turmeric (*Curcuma longa*): It has long been a cornerstone of Ayurvedic medicine, valued particularly for its potent anti-inflammatory and antioxidant properties. Its major bioactive constituents-curcuminoids, including curcumin, demethoxycurcumin, and bisdemethoxycurcumin-have been extensively studied for their therapeutic potential in cancer [56]. Curcumin, the most prominent curcuminoid, exerts broad-spectrum anticancer effects by suppressing multiple cellular processes involved in tumour initiation and progression, such as cellular transformation, uncontrolled proliferation, invasion, angiogenesis, and metastasis. These effects are largely mediated through the inhibition of TNF-α-induced NF-κB activation and subsequent downregulation of NF-κB-dependent genes implicated in cell survival, anti-apoptotic signalling, and metastatic behaviour [56,57]. In addition to these well-characterized mechanisms, curcumin has also been reported to induce oxidative stress and DNA damage in cancer cells, accompanied by the downregulation of critical DNA repair proteins such as BRCA1 and RAD51. This disruption of homologous recombination repair potentially sensitizes tumour cells to PARP inhibitors, providing a rationale for combinatorial strategies in cancers with compromised DNA repair capacity [43,54]. Furthermore, preclinical studies have demonstrated that curcumin can enhance the cytotoxic effects of PARP inhibitors, suggesting a functional connection to ADPr signalling, a key regulatory pathway in DNA repair and cell death [43].

Kalmegh (*Andrographis paniculata*): It is a well-known Ayurvedic herb with documented anticancer properties. Its principal bioactive compound, andrographolide, exhibits strong anti-inflammatory, antioxidant, and immunomodulatory activities, contributing to its ability to inhibit cancer cell proliferation, induce apoptosis, and suppress tumour progression in breast, prostate, lung, and colorectal cancers [58]. Kalmegh extracts have also been shown to enhance conventional therapies and reduce chemotherapy-associated toxicity [58]. Emerging evidence indicates that andrographolide induces DNA damage and oxidative stress [59], both of which activate PARP1, a key enzyme in ADPr signalling and DNA repair [34]. Though direct effects on ADP-ribosyl transferases are yet to be clarified, the modulation of upstream pathways such as PI3K/AKT suggests a potential influence on ADPr-mediated cell death and repair mechanisms [60].

Ashwagandha (*Withania somnifera*): The plant *Withania somnifera*, commonly known as Ashwagandha or Indian ginseng, holds a prominent place in Ayurvedic medicine for its anti-inflammatory and antitumor properties. Its bioactive constituents, particularly withanolides derived from roots and leaves, exhibit strong anticancer effects by suppressing NF-κB activation, inhibiting cell proliferation, enhancing apoptosis, and reducing invasion in various cancer models [61]. Among these, withaferin A has shown notable antitumor activity through the inhibition of Notch-1 signalling and downregulation of pro-survival pathways such as Akt/NF-κB/Bcl-2, highlighting its therapeutic potential [62]. In addition to these effects, withaferin A has been reported to induce endoplasmic reticulum (ER) stress, activate caspases, and downregulate survival signals like NF-κB-mechanisms that intersect with PARP1 activation and ADPr-dependent cell death pathways [63,64]. Furthermore, withaferin A can cause DNA fragmentation and oxidative damage, potentially leading to PARP1 overactivation and NAD^+^ depletion-hallmarks of parthanatos, a regulated necrotic cell death driven by excessive ADPr [35].

Giloy (*Tinospora cordifolia*): It is an herbaceous vine native to the Indian subcontinent, is widely revered in Ayurvedic medicine for its broad therapeutic potential, including its role in cancer management [65,66]. Giloy is believed to exert anticancer effects through its immunomodulatory, antioxidant, and anti-inflammatory properties. Studies have demonstrated that extracts from Giloy can inhibit the proliferation of cancer cells and induce apoptosis in various cancer types, including breast, colon, and liver cancers [65]. Moreover, Giloy has been shown to enhance the efficacy of chemotherapy and radiotherapy, while reducing their toxicity, highlighting its value as a complementary therapy in oncology [65,66]. Recent findings also suggest that oxidative stress modulation by Giloy may intersect with ADPr signalling pathways. Since ROS-induced DNA damage is a known activator of PARP1, key to initiating ADPr-mediated DNA repair and cell death mechanisms, the antioxidant and pro-apoptotic effects of Giloy raise the possibility of indirect regulation of this pathway [34]. Although direct evidence linking *T. cordifolia* constituents to PARP or ADP-ribosyl transferase activity is currently lacking, its influence on upstream events such as DNA damage response and immune signalling suggests potential crosstalk with ADPr-dependent processes, warranting further investigation.

Neem (*Azadirachta indica*): It is commonly known as neem, has long been used in traditional medicine for its broad therapeutic potential. Phytochemicals isolated from neem leaves, such as azadirachtin and nimbolide, exhibit significant anticancer properties. Azadirachtin has been shown to act as an anti-tumor agent by inhibiting NF-κB, a central regulator of cancer cell survival and progression [67]. Nimbolide, a limonoid found in neem leaves and flowers, has demonstrated potent effects against prostate cancer, primarily through targeting androgen receptor signalling, generating reactive oxygen species (ROS), and inducing cell cycle arrest and DNA damage [68]. These actions suggest a potential interface with PARP-mediated DNA damage responses, as ROS and DNA damage are upstream activators of ADPr signalling. While the direct modulation of PARP enzymes by nimbolide has yet to be fully elucidated, its ability to induce genotoxic stress supports a plausible link to ADPr-dependent cancer cell death mechanisms.

Amalaki (*Phyllanthus emblica*): It is also known as Indian gooseberry or Amla, has gained attention for its potential anticancer properties. Research suggests that compounds found in Amalaki-such as polyphenols, flavonoids, and tannins-exhibit antioxidant and anti-inflammatory effects that may contribute to its anticancer activity [33,69]. Additionally, Amalaki extracts have shown cytotoxic effects on cancer cells, inhibiting their growth and proliferation, and are believed to enhance the immune system, which can aid in combating cancer [33,69]. Although direct evidence is limited, Amalaki may influence ADPr-associated pathways via its effects on oxidative stress, DNA damage, and NAD^+^ metabolism.

Tulsi (*Ocimum sanctum*): It demonstrates notable anticancer potential primarily through its antioxidant properties. Ursolic acid, a key bioactive compound in tulsi, inhibits NF-κB activation induced by various carcinogens, thereby downregulating genes such as cyclin D1, COX-2, and MMP-9 involved in cancer progression [33,70]. Additionally, it suppresses STAT3 activation, which is crucial for cancer cell survival and proliferation, and promotes apoptosis and cell cycle arrest, thereby enhancing the effects of other anticancer agents [70,71]. Tulsi’s ability to modulate redox balance, inflammation, and transcriptional responses suggests a possible indirect influence on ADPr-dependent pathways relevant to cancer and immunity [71,72]. The dualistic nature of its action-either suppressive or promotive-warrants further investigation to clarify its role in cancer biology [73]. Overall, its broad pharmacological profile makes tulsi a promising candidate for cancer prevention and therapy [33,70].

Punarnava (*Boerhaavia diffusa*): It is commonly known as Punarnava, has long been valued in traditional medicine for its wide range of therapeutic properties [74,75]. Recent studies have highlighted its anti-cancer potential. The aqueous methanolic extract of B. diffusa significantly reduced metastasis formation in B16F-10 melanoma models, while punarnavine, an alkaloid isolated from the plant, enhanced immune responses against metastatic progression in mice [33,76]. In addition, Punarnava contains bioactive phytochemicals such as boeravinone B and punarnavine, which exhibit strong antioxidant and anti-inflammatory activities [77]. Given that oxidative stress and DNA damage are key triggers for PARP activation, these compounds may indirectly modulate ADPr by mitigating ROS levels. Furthermore, B. diffusa has shown genoprotective effects, including a reduction in cyclophosphamide-induced chromosomal aberrations, suggesting a possible role in DNA repair processes—though direct evidence linking it to ADPr remains to be established [78].

Vasicinone (*Adhatoda vasica*): Vasicinone, derived from Adhatoda vasica Nees, shows bronchodilator activity and potential anti-proliferative effects on cancer cells. Research on A549 lung carcinoma cells demonstrated reduced viability, DNA fragmentation, LDH leakage, disrupted mitochondrial potential, and impaired wound healing upon vasicinone treatment [79]. Annexin V/PI staining suggested apoptosis induction via compromised membrane integrity. Mechanistically, vasicinone downregulated Bcl-2 and Fas death receptor while upregulating PARP, BAD, and cytochrome c, implicating Fas death receptor and Bcl-2 signalling in apoptosis [79]. Moreover, vasicinone exhibited antioxidant properties, reducing ROS levels and scavenging free radicals, suggesting its potential as a therapeutic agent against oxidative stress-induced lung cancer [33,79]. Understanding the specific PARP involved in the apoptosis of A549 lung carcinoma cells in response to vasicinone treatment remains unexplored. The involvement of PARP-induced cellular ADPr by vasicinone represent a promising avenue for Ayurvedic research. Exploring into this field can shed light on the connection between Ayurvedic components and cancer, particularly lung cancer. Investigating how vasicinone influences PARP activity and subsequent ADPr processes may unravel novel therapeutic strategies rooted in Ayurveda for combating lung cancer. This exploration holds significant potential for advancing our comprehension of the molecular structures underlying the anti-cancer properties of Ayurvedic compounds like vasicinone (Figure 2) [79].

## 3. Phytochemical-Induced Poly-ADP-Ribosylation in Carcinogenesis: Insights from Ayurvedic Thymus Species

Poly-ADPr (PARylation), a post-translational modification catalysed primarily by PARPs, plays a pivotal role in cellular processes such as DNA repair, chromatin remodelling, transcriptional regulation, and programmed cell death [1,2]. Dysregulation of PARylation is associated with genomic instability, impaired DNA damage response, and aberrant gene expression, contributing to carcinogenesis and tumour progression [1,2,80]. Although extensive evidence from molecular and animal model studies implicates PARPs in cancer development, their precise role in human malignancies remains incompletely defined [80].

While direct links between Ayurvedic phytochemicals and ADPr signalling remain limited, several indirect mechanistic cues suggest a plausible intersection. ADPr is central to cellular stress responses, encompassing DNA damage signalling, apoptosis, and immune regulation, which are hallmarks of cancer initiation and progression [34,35]. Certain phytochemicals derived from Ayurvedic herbs, such as curcumin (*Curcuma longa*), withaferin A (*Withania somnifera*), and nimbolide (*Azadirachta indica*), are known to influence upstream regulators of PARP activation, including oxidative stress, redox homeostasis, and NF-κB signalling [42,63,68]. For instance, curcumin has been shown to sensitize cancer cells to PARP inhibitors by enhancing oxidative DNA damage and impairing homologous recombination repair [43]. Withaferin A similarly induces ER stress and apoptosis, events that converge on PARP1 activation during cell death [64]. These observations suggest that phytochemicals may modulate ADPr signalling directly through PARP inhibition or indirectly via NAD^+^ metabolism and stress-response pathways (Figure 2). However, experimental validation is needed to elucidate these interactions and assess the therapeutic potential of combining Ayurvedic agents with PARP inhibitors in DNA repair-deficient cancers. It has been reported that few phytochemicals directly inhibit PARP enzymes, notable examples include epigallocatechin-3-gallate (EGCG), which inhibits PARP16 (IC_50_ ≈ 14.5 µM), and resveratrol, which suppresses PARP1 activity at micro- to sub-micromolar levels. Flavonoids such as quercetin modulate NAD^+^ metabolism and DNA damage response (DDR) pathways, sensitizing cells to PARP inhibition, though quantitative enzymatic data remain scarce [81]. These gaps highlight the need for systematic enzymatic and structure–activity studies to validate phytochemical–PARP interactions and assess their therapeutic potential in DNA repair-deficient cancers.

Investigations into PARP gene expression and structure have also uncovered important oncogenic associations. Knockout and transgenic models for PARP-1, PARP-2, and PARG have deepened understanding of PARylation’s role in tumour biology [80]. PARP-1 is crucial for base excision repair, chromosomal stability, and epigenetic regulation. Its dysregulation may lead to cell death via NAD^+^ depletion, potentially selecting for PARP-1-deficient clones with a growth advantage during tumour progression [82]. Elevated PARP-1 expression and activity have been reported in Ewing’s sarcoma cell lines, linked to ETS-1 activation [83]. Conversely, weak PARP-1 expression is associated with heightened genomic instability in breast cancer, often coinciding with gene amplification at chromosome 1q41–42 [84]. Additionally, reduced poly (ADP-ribose) synthesis in lymphocytes from bleomycin-treated laryngeal cancer patients suggests that low PARP activity may elevate cancer risk [85,86]. In gastric cancer cells, structural alterations in the PARP-1 gene have been observed, although their functional consequences remain unclear. Emerging evidence also implicates PARP-2 and PARP-3 in carcinogenesis, with PARP-3 located at chromosome 3p21.1-3p21.31-a region frequently affected by loss of heterozygosity in early-stage lung cancer [87].

Ayurvedic medicine has recently attracted attention for its potential to modulate ADPr pathways [88]. Dutta et al. [26] reviewed the anticancer potential of *Withania somnifera* (Ashwagandha), emphasizing its efficacy in breast, colon, prostate, ovarian, lung, and brain cancers. Withaferin A (WFA), its bioactive component, demonstrated cytotoxicity in preclinical models and was well-tolerated in patients. Synergistic antitumor effects were reported when WFA was combined with Sorafenib in papillary and anaplastic thyroid cancers, resulting in PARP and caspase-3 cleavage, downregulation of BRAF/Raf-1, and HSP inhibition [89]. Curcumin also exhibits strong modulatory effects on DNA damage response (DDR) pathways, including homologous recombination, non-homologous end joining (NHEJ), and G2/M checkpoint regulation. Ogiwara et al. [27] showed that curcumin targets CBP/p300 HATs and ATR kinase, sensitizing DDR-competent cells to PARP inhibitors and promoting mitotic catastrophe. Despite promising findings, the precise molecular mechanisms underlying the action of traditional Ayurvedic herbs-such as turmeric, ashwagandha, amalaki, giloy, and kalmegh-on ADPr signalling remain largely undefined [90]. Few studies have directly investigated the link between Ayurvedic herbs and PARP-mediated ADPr [26,27], highlighting a critical gap. Future studies focusing on cellular ADPr profiling in response to Ayurvedic extracts could unveil novel mechanistic insights into cancer modulation. This could open new avenues for rationally designed phytochemical-based therapies targeting ADPr signalling in malignancies.

## 4. Phytochemicals in p53 Signalling and ADP-Ribosylation Pathways

p53 functions as a master regulator of cellular stress responses and is closely intertwined with ADPr signalling. PARP1 and related PARP family enzymes directly ADP-ribosylate p53, thereby modulating its stability, transcriptional activity, and control over cell cycle arrest and apoptosis [91]. While this review primarily focuses on phytochemicals associated with PARP- and ADPr-mediated cancer regulation, p53 emerges as a key downstream effector integrating these pathways with cell fate decisions. Notably, Ayurvedic phytochemicals such as andrographolide, curcumin, resveratrol, and withaferin A have been reported to influence p53 signalling—often through DNA damage and oxidative stress responses that indirectly engage PARP1 [92,93]. p53 is both functionally and biochemically linked to PARP1/ADPr, and these phytochemicals can influence PARP1 activity or p53-mediated pathways [93]. For example, resveratrol inhibits PARP1 activity and interferes with its nucleosome binding and catalytic functions at micromolar concentrations [94]. A low-dose combination of withaferin A and caffeic acid phenethyl ester activates p53 and produces effects similar to PARP1 inhibition, promoting growth arrest and apoptosis in cancer models [95]. Curcumin has been shown to induce oxidative stress and DNA damage, upregulate p53, and interact with DNA damage response pathways, modulating PARP-related responses and sensitizing cells to PARP inhibitors. Notably, curcumin-induced apoptosis in colon cancer cells can occur independently of p53 status, highlighting its potential for treating tumours resistant to conventional therapies due to p53 defects [96]. Andrographolide, in combination with TRAIL, inhibits growth, decreases proliferation, reduces colony formation, suppresses migration, and promotes caspase-mediated apoptosis in T24 bladder cancer cells. This sensitization occurs via upregulation of death receptors (DR4 and DR5) in a p53-dependent manner, while also inactivating NF-κB signalling through transcriptional downregulation of p65/RelA, enhancing TRAIL-mediated cytotoxicity [97]. Furthermore, inhibition of poly (ADP-ribosyl) ation has been shown to increase p53 levels and activation in certain contexts, demonstrating functional crosstalk between PARP activity and p53 signalling [98]. This interplay implies that ADPr may act as a molecular bridge modulating phytochemical-driven activation or stabilization of the p53 signaling network. Understanding this connection could deepen current insights into how natural compounds restore tumor suppressor functions and influence DNA repair, apoptosis, and stress responses. Future studies employing ADPr profiling in cells treated with Ayurvedic extracts may uncover novel mechanistic layers of cancer modulation. Such findings could pave the way for rationally designed phytochemical-based therapeutics targeting ADPr-associated signaling in malignancies [91].

## 5. Phytochemical-Induced Coupling of Cellular Stress and the ADPr Axis in Apoptotic and Autophagic Cell Death

Phytochemicals regulate cancer cell death via apoptotic and autophagic pathways. They disrupt mitochondrial function, induce ER stress, and modulate cell cycle progression [99]. Some promote apoptosis-autophagic cell death by modulating mitochondrial biogenesis and microRNA expression. Additionally, phytochemicals activate autophagic signalling, inhibiting cell growth, and promoting autophagy for cell death. Curcumin elevates ROS and DNA damage in cancer cells while inducing autophagy through ERK1/2 and p38 MAPK phosphorylation, and Akt and P54 JNK inhibition [100]. Quercetin exhibits anticancer effects in primary effusion lymphoma (PEL) by reducing cytokine release and inhibiting PI3K/Akt/mTOR and STAT3 pathway-induced autophagy, leading to PEL cell death [101]. Cucurbitacin B promotes DNA damage via γH2AX protein expression and ROS generation, inducing autophagy in MCF-7 cells [102].

The Bcl-2 family of proteins regulates programmed cell death through the balance between pro-apoptotic members (Bax, BAD) and anti-apoptotic members (Bcl-2, Bcl-xl). Dysregulation of this balance disrupts mitochondrial membrane potential, leading to cytochrome C release and formation of the apoptosome complex with Apaf-1 and procaspase-9, thereby activating caspase-3 [103,104]. Ayurvedic plant-derived phytochemicals have been shown to modulate these apoptotic cascades by altering mitochondrial potential, increasing annexin-V/PI-positive apoptotic cells, and upregulating mRNA and protein expression of BAD, cytochrome C, and caspase-3, while downregulating Bcl-2 [79]. In lung cancer models, vasicine specifically enhances PARP activation, further amplifying apoptosis [79]. Additionally, key phytochemicals induce ER stress, trigger cell-cycle arrest, and activate autophagic signaling, promoting programmed cell death and inhibiting tumor progression [99].

**Figure 3:**
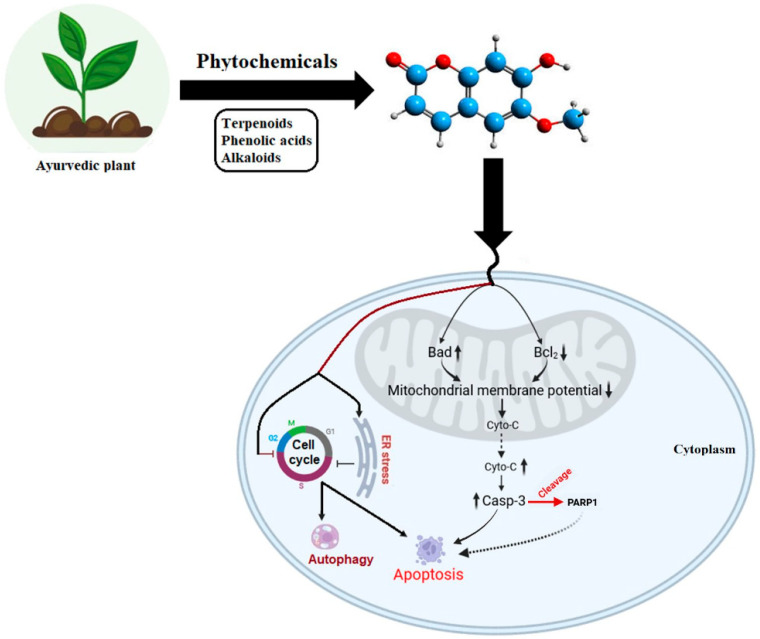
Molecular targets of Ayurvedic phytochemicals. The schematic illustrates the potential mechanisms by which Ayurvedic plant-derived phytochemicals activate apoptotic and autophagic pathways in human carcinoma cells. Treatment with Ayurvedic compounds upregulates pro-apoptotic markers such as BAD, cytochrome C, and caspase 3 (indicated by the upward arrow), while downregulating the anti-apoptotic protein Bcl-2 (indicated by the downward arrow). Activation of PARP further amplifies apoptotic signaling (indicated by the curved dotted arrow), as observed in lung cancer cells treated with vasicine [79]. These phytochemicals modulate signal transduction networks governing ER stress, cell-cycle arrest, and autophagy, promoting programmed cell death and inhibiting tumor growth [99].

Research demonstrated that Oridonin induces apoptosis in human carcinoma BEL-7402 cells via caspase-3 activation and downregulation of Bcl-2 expression, accompanied by Bax upregulation [105]. Rottlerin treatment in breast cancer stem cells (CSCs) inhibits Akt and mTOR phosphorylation while promoting AMPK phosphorylation, leading to apoptosis [106]. Glucosinolate-derived phenethyl isothiocyanates (PEITC) downregulate HER2, EGFR, and STAT3 expression and induce apoptosis in mice via caspase 3 and PARP cleavage. Additionally, NCTD demonstrates anticancer properties by inhibiting c-Met and mTOR [107,108]. Furthermore, PARP is known to play a crucial role in DNA damage and repair processes [109,110,111]. Overexpression of PARP can initiate apoptosis by promoting the release of mitochondrial apoptosis-inducing factors and inducing double-stranded DNA breaks. Studies on vasicine treatment in lung cancer cells have shown an increase in both mRNA and protein expression of PARP, along with DNA fragmentation [79]. This suggests that the PARP-mediated ADP ribosylation pathway could be an interesting area for future research, particularly in understanding cellular biomolecule modifications induced by Ayurvedic treatments (Figure 3).

## 6. ADP-Ribosylation and Inflammatory Pathways: Insights from Ayurvedic Medicine

ADPr, particularly PARylation, plays a critical role in modulating inflammation by influencing immune signalling pathways, cytokine production, and cell death processes. Among the PARP family, PARP1 is a central regulator of inflammatory responses. Its activation enhances transcription of pro-inflammatory genes through interactions with key transcription factors such as NF-κB and AP-1 [112]. However, excessive or prolonged PARP1 activation can lead to NAD^+^ and ATP depletion, resulting in cellular dysfunction and death-an underlying mechanism implicated in a variety of inflammatory diseases [113]. Notably, pharmacological inhibition of PARP has demonstrated anti-inflammatory efficacy in preclinical models of sepsis, arthritis, and colitis, supporting its potential as a therapeutic target in chronic inflammatory conditions [114,115].

Inflammation is increasingly recognized as a central link between metabolic disorders and cancer. Disruptions in immune regulation, redox balance, and cellular homeostasis not only promote inflammatory diseases but also create a tumour-supportive microenvironment [116]. Chronic inflammation, whether driven by genetic mutations (intrinsic pathway) or environmental stimuli (extrinsic pathway), contributes to the initiation and progression of malignancies. Key inflammatory mediators-including NF-κB, STAT3, and HIF-1α-activate transcription of pro-inflammatory genes such as cytokines, chemokines, COX-2, prostaglandins, iNOS, and nitric oxide. This cascade promotes the recruitment of tumour-associated macrophages (TAMs) and other immune cells into the tumor microenvironment, reinforcing inflammation and aiding tumour progression [117,118].

Mechanistically, reactive oxygen and nitrogen species (ROS/RNS) induce inflammatory responses by damaging DNA, lipids, and proteins, leading to gene mutations and the formation of advanced glycation end products (AGEs). These AGEs interact with their receptor (RAGE), activating NF-κB at sites of tissue injury and bypassing natural anti-inflammatory mechanisms, thereby perpetuating chronic inflammation [119,120]. Consequently, anti-inflammatory interventions-such as nonsteroidal anti-inflammatory drugs (NSAIDs) and selective COX-2 inhibitors-have shown efficacy in both suppressing tumour growth and reducing oxidative stress and angiogenesis [121]. In particular, blocking prostaglandin E2 (PGE2) production via inhibition of iNOS and COX-2 is emerging as a promising strategy for inflammation-associated cancer therapy.

Several Ayurvedic phytochemicals with established anti-inflammatory properties may intersect with ADPr pathways. For instance, *Bhadradarvadi Kashayam* modulates the NF-κB pathway, highlighting its potential as a natural anti-inflammatory agent [122,123,124]. Likewise, compounds such as curcumin (*Curcuma longa*), epigallocatechin gallate (EGCG) from green tea, resveratrol from grapes, and guggulsterone from *Commiphora wightii* exhibit potent anti-inflammatory and anticancer activities, partly through suppression of NF-κB-dependent transcription. Among these, guggulsterone stands out for its ability to induce apoptosis, inhibit tumor invasion, angiogenesis, and metastasis, and modulate STAT3 signaling [125]. These phytochemicals may influence inflammatory and ADPr-associated signaling, offering promising prospects for integrated cancer therapeutics. Future studies investigating their role in ADPr regulation could unveil novel anti-inflammatory mechanisms grounded in Ayurvedic medicine.

## 7. Integrative Cancer Therapies: ADP-Ribosylation in Ayurvedic and Allopathic Perspectives

Entire medical systems such as Ayurveda operate independently of biomedicine, drawing upon canonical texts and holistic, individualized therapeutic strategies [126,127]. While allopathic medicine effectively manages acute and life-threatening conditions, its symptom-centric approach often results in adverse side effects and limited long-term efficacy. In contrast, Ayurvedic formulations have been investigated for their potential molecular and therapeutic effects, particularly in chronic diseases such as hemiplegia, haemorrhoids, and arthritis, where conventional therapies remain inadequate [128,129]. Emerging studies suggest that these formulations exert beneficial effects through the modulation of inflammatory, oxidative stress, and metabolic signaling pathways, thereby promoting cellular homeostasis and tissue repair. Rooted in centuries of empirical wisdom, Ayurveda offers an integrative and mechanistically relevant approach to disease modulation beyond conventional pharmacotherapy [130,131].

An emerging approach in cancer management emphasizes holistic systems such as Ayurveda [132,133], which views cancer as a tridoshic imbalance involving vata, pitta, and kapha [131]. Ayurvedic care aims to restore systemic balance, enhance resilience, and mitigate the adverse effects of biomedical therapies. It recognizes genetic, dietary, and lifestyle factors as major contributors, paralleling modern perspectives [134,135,136]. The roles of kapha in tissue growth, pitta in malignant transformation, and vata in metastasis are central, with *granthi* and *arbuda* considered traditional analogues of cancer [131].

Recent advances in molecular biology have opened up new possibilities for decoding the therapeutic effects of Ayurvedic medicine by linking them to conserved cellular signalling pathways. One such promising link lies in ADPr, which plays crucial roles in DNA damage repair, transcriptional regulation, cell death, and importantly, immune modulation [112,113]. Inflammation-associated PARP1 activation is well-documented in both acute and chronic disease contexts, including cancer [112,113]. Sustained PARP1 activation leads to NAD^+^ depletion and mitochondrial dysfunction, contributing to inflammatory and tumour-promoting microenvironments [114]. Notably, emerging studies indicate that several phytochemicals used in Ayurvedic formulations-such as curcumin, resveratrol, and rosmarinic acid modulate ADPr by either inhibiting PARPs or affecting the expression of PAR-regulated genes, suggesting a plausible mechanistic bridge between Ayurvedic interventions and molecular oncology [137,138,139]. Despite the standardized, evidence-based framework of allopathic medicine, alternative medical systems continue to provide diverse therapeutic perspectives with varying degrees of validation [4,131]. A regional survey by Choi et al. involving nearly 900 participants from Southeast Asia revealed that more than half preferred traditional and complementary medicine (T&CM), particularly Ayurveda, for cancer care. The study emphasized integrating traditional and biomedical approaches to improve patient outcomes [140].

Allopathic cancer management employs surgery, radiotherapy, chemotherapy, immunotherapy, hormonal therapy, and targeted therapeutics, supported by recent advances in nanotechnology, RNA profiling, and CRISPR-mediated interventions [141,142]. While its strong empirical and regulatory foundation ensures credibility, alternative systems often lack similar standardization and reproducibility, which constrains their broader acceptance [131,143].

Ayurveda offers a distinct yet complementary paradigm, focusing on detoxification, immune modulation, and the restoration of systemic equilibrium through *Dosh*, *Dhatu*, *Mal*, *Upadhatu*, and *Oaj* regulation [4,131]. Mechanistically, emerging evidence suggests that Ayurvedic phytochemicals influence redox homeostasis, inflammatory signaling, and NAD^+^ metabolism—processes tightly linked to ADPr dynamics. Since ADPr governs DNA repair, metabolic adaptation, and cell death pathways, its modulation by plant-derived bioactives could underpin the anticancer efficacy of Ayurvedic formulations. Thus, Ayurveda’s holistic framework, when integrated with molecular oncology, may enable rationally designed combinatorial therapies targeting ADPr-mediated signaling networks in cancer.

## 8. Phytochemical–Nanoparticle Coupling and Targeted Delivery Strategies for Modulating ADP-Ribosylation Pathways in Cancer

In the context of Ayurvedic-inspired nanocarriers, it is noteworthy that classical formulations such as the *bhasma* preparations from the Rasashastra tradition reflect several fundamental principles of modern nanoscale delivery systems. *Bhasmas* are produced through repeated purification (*śodhana*) and incineration (*māraṇa*) processes, yielding metal or mineral particles in the nanometre range (typically 5–50 nm) that are highly absorbable and bio-assimilable [144,145]. These preparations exhibit physicochemical properties analogous to contemporary nanocarriers-such as high surface area, enhanced systemic distribution (“*śighra vyāpī*”), micro-dose efficacy (“*alpamātra*”), and targeted delivery behaviour (“*yogavāhī*”)—demonstrating their advanced pharmacological rationale [144,146]. By highlighting the nanoscale essence of *bhasmas*, this perspective emphasizes how Ayurvedic carrier-based concepts provide both a philosophical and mechanistic foundation for modern Phyto-nanomedicine design. Nanotechnology thus represents a natural extension of these ancient principles, offering new opportunities for diagnostic and therapeutic innovations. *Bhasmas* can be viewed as early prototypes of nanoparticle-based delivery, following a traditional “top-down” synthesis approach, and their future standardization should integrate modern nanotechnological characterization methods [144].

The integration of nanotechnology with Ayurvedic phytochemicals has opened a promising avenue for enhancing bioavailability, specificity, and efficacy of natural compounds in cancer therapy [147,148]. A growing body of research suggests that nano formulations of phytochemicals not only improve solubility and pharmacokinetics but also enable precision targeting of critical oncogenic pathways such as ADPr, particularly those mediated by PARPs. PARPs, especially PARP1 and PARP2, play a pivotal role in DNA repair, chromatin remodelling, and cellular stress responses [149]. Dysregulation of PARP activity is a hallmark of several cancers, and PARP inhibitors (PARPi) like Olaparib have already received FDA approval [150,151]. However, synthetic inhibitors often suffer from toxicity, resistance, and limited tumour selectivity. This has stimulated interest in plant-derived modulators as natural alternatives or adjuvants.

Phytochemicals such as curcumin, resveratrol, quercetin, and rosmarinic acid possess documented PARP-inhibitory or modulatory effects. Their nano-encapsulation, using liposomes, PLGA (poly-lactic-co-glycolic acid) nanoparticles, solid lipid nanoparticles (SLNs), or gold nanoparticles (AuNPs), has shown promise in targeting PARP-dependent signalling and DNA repair pathways in preclinical cancer models [55,152]. For instance, curcumin-loaded nanoparticles have demonstrated PARP1 suppression in models of breast and colorectal cancer, enhancing DNA damage and apoptotic cell death when combined with ionizing radiation or chemotherapeutics [152]. Similarly, resveratrol-loaded PLGA nanoparticles have improved cellular uptake and induced DNA damage through modulation of PARP cleavage in glioma and ovarian cancer models [153]. In another example, quercetin-conjugated gold nanoparticles were shown to inhibit PARP1 activity in prostate cancer cells, amplifying DNA damage and reducing cell viability more effectively than free quercetin [154]. Mechanistically, the crosstalk between phytochemical-induced oxidative stress and the PARP axis is central to these outcomes. Many polyphenols induce mitochondrial dysfunction and ER stress, leading to single strand breaks and consequent PARP activation. Overactivation of PARP can cause cellular energy crisis and necrosis, which, when precisely modulated by phytochemicals, may tilt the balance toward apoptotic cell death in cancer cells while sparing healthy tissues [155,156].

While Ayurvedic medicine-based nanotechnology in cancer therapy faces significant challenges, including issues of standardization, potential toxicity, and translational bottlenecks [157,158,159], these obstacles can be systematically addressed through the application of green nanofabrication techniques, rigorous analytical characterization, and robust preclinical validation. Additionally, the integration of multidisciplinary approaches, including the design of engineered nanoparticles for cancer vaccination and immunotherapy, further enhances the safety, specificity, and therapeutic potential of these nano formulations [160,161,162]. The convergence of traditional Ayurvedic principles with modern nanoscience thus holds the promise of establishing a rational, scientifically credible, and translationally viable foundation for next-generation anticancer therapeutics.

Recent efforts have increasingly focused on scaling these preclinical findings toward translational endpoints. Several nano-formulated phytochemicals are currently being evaluated in early-phase clinical trials or investigational studies. Table 1 [163,164,165,166,167,168,169,170,171,172,173,174,175,176,177,178,179] summarises representative examples of phytochemical–nanoparticle conjugates, their target cancer types, and associated PARP-driven mechanistic insights. For instance, nano-curcumin, which indirectly modulates PARP1-mediated responses through oxidative stress pathways, has already completed multiple Phase I/II trials in cancers such as pancreatic and colorectal carcinoma [180,181]. Another promising direction is the development of polyphenol-based nano-drug delivery systems designed to co-deliver phytochemicals alongside PARP inhibitors, thereby enhancing synergistic anti-tumour effects while minimizing toxicity. These combinatorial platforms mark a paradigm shift in dual-targeting strategies-integrating the wisdom of traditional Ayurveda with the precision of modern molecular oncology. Looking ahead, rigorous exploration of Ayurvedic phytochemical–nanoparticle formulations in the context of ADPr signalling remains essential. High-throughput screening of ADPr-related endpoints, imaging-guided biodistribution analyses, and computational modelling of phytochemical–PARP interactions could further accelerate progress. As clinical investigations mature, such natural compound-based nanomedicines hold the potential to emerge either as standalone therapeutic agents or as sensitizers augmenting existing DNA repair-targeted regimens [182,183].

## 9. Concluding Reflections

Ayurvedic phytochemicals mediate two distinct cellular pathways: one linked to organelle-induced stress responses involving mitochondria and the endoplasmic reticulum that culminate in apoptotic signalling (Figure 3), and another potential mechanism in which such stress may trigger DNA damage, particularly in cancer cells. This can activate DNA damage response pathways, including the spatiotemporally regulated activity of PARPs, influencing ADPr-driven survival or death outcomes (Figure 2). Although our proposed model (Figure 2) outlines these processes, detailed investigations are still required to understand how phytochemical-induced stress determines cell fate through ADPr. This review integrates traditional Ayurvedic principles with modern biomedical evidence to provide molecular insights into cancer progression and therapy. It highlights how phytochemicals influence signalling networks associated with apoptosis, stress responses, and genomic stability. The synthesis of literature reveals phytochemicals as promising agents that bridge ancient wisdom and molecular oncology.

A major highlight is the identification of key research gaps that limit translational progress. While single-compound studies offer mechanistic clarity, they often face limitations such as resistance and poor pharmacokinetic behaviour. In contrast, whole-plant extracts or multi-compound formulations central to Ayurvedic practice may offer synergistic efficacy, better bioavailability, and reduced toxicity [33]. Integrating Ayurvedic constitutional types (Prakriti: Vata, Pitta, Kapha) with genomic and molecular data also presents opportunities for personalized cancer management [4,184]. Such approaches reinforce Ayurveda’s relevance in precision medicine and therapeutic innovation.

## 10. Future Perspectives

Future studies should focus on decoding how phytochemicals influence genome stability, particularly through interactions with DNA repair pathways and redox regulation. Many natural compounds affect chromatin remodelling, repair protein recruitment, and oxidative signalling, offering therapeutic potential in cancers driven by genomic instability [80,185]. The psychosomatic component of cancer also warrants greater attention. Chronic stress and depression influence cancer initiation, progression, and prognosis through activation of the hypothalamic–pituitary–adrenal axis and sympathetic signalling [186,187,188]. Ayurvedic lifestyle interventions including diet, daily routines, yoga, breathing practices, and meditation can serve as non-pharmacological approaches to reduce stress-induced cancer risk and improve overall outcomes.

Advances in molecular biology such as CRISPR/Cas9, ZFNs, and TALENs have enhanced the precision of gene targeting [143]. Combining gene-editing with phytochemical modulation could generate innovative therapeutic strategies. Bioactive compounds including alkaloids, tannins, saponins, phenolics, and flavonoids continue to demonstrate antiproliferative, antioxidant, and pro-apoptotic activity [189]. For example, Vasicine from *Adhatoda vasica* shows selective cytotoxicity toward A549 lung cancer cells while sparing normal fibroblasts, underscoring its potential as an anticancer candidate [79,190]. Despite promising outcomes, clinical translation remains challenging due to issues of solubility, stability, and bioavailability. Nanocarrier-based delivery systems offer a way forward by improving stability, controlled release, and targeted delivery of phytocompounds [191]. Integrating Ayurvedic phytochemicals with nanotechnology and exploring their influence on ADPr and PARP pathways could open new avenues in precision oncology and immunomodulation.

Future research should prioritize the mechanistic validation of phytochemical–ADPr interactions, the establishment of standardized nano formulation protocols, comprehensive safety assessments, and well-structured clinical trials. Of particular interest is the exploration of how Ayurvedic phytochemicals influence p53 regulation, Sirtuin-mediated ADPr, and upstream–downstream signalling hierarchies that dictate cell fate decisions. Bridging traditional pharmacology with nanomedicine and molecular oncology will be pivotal for developing integrative, sustainable, and effective therapeutic approaches [4,33,131,184].

In summary, advancing the scientific foundation of Ayurveda through molecular-level inquiry and technological innovation will not only refine our understanding of cancer biology but also guide the evolution of inclusive and precision-based cancer care for the future.

## Figures and Tables

**Table 1:** Potential Phytochemical–Nanoparticle Coupling Strategies Targeting ADP-ribosylation Pathways in Cancer.

Sl No.	Name of the Phytochemical	Deliverable Phytochemical–Nanoparticle Conjugate	Size of the Formulation (nm)	Zeta Potential Values (mV)	Mechanistic Insights: ADPr/PARP Pathway	Cancer Type(s) Studied	Clinical Status	References
1	Rosamarinic acid	Rosmarinic acid titanium oxide and selenium-doped graphene oxide nanoparticles (rosamarinic acid@Se-TiO_2_-GO nanocomplex)	344.8 ± 43.2	−33.1 ± 2.64	Cleaved PARP-1, via p53 upregulation induced by HDAC2 downregulation, promoted apoptosis.	Prostate cancer	Preclinical in vitro only	[163]
2	Resveratrol	Liposomal formulation	-	-	Synergizes with PARP inhibitors, modulates DNA repair pathways.	Breast, prostate	Preclinical	[164]
3	Andrographolide	Solid Lipid Nanoparticles (ADG-SLNs)	286.1 ± 8.03	−20.5 ± 0.3	ADG-SLNs enhance apoptosis in HN models; PARP/ADPr involvement (cleaved PARP-1, PARylation) remains to be tested.	Head-and-neck cancer (HIOEC, Leuk-1, HN6, HN30 cells)	Preclinical in vitro only	[165]
4	Curcumin	Poly (lactic acid-co-glycolic acid) (PLGA) curcumin (Nano-CUR)	70 ± 3.9	-	Enhances PARP inhibition, increases DNA damage, induces apoptosis.	Breast, ovarian	Preclinical	[166]
5	Quercetin	Solid lipid nanoparticles	154	−27.7	Inhibits PARP activity, enhances chemosensitivity.	Lung, colon cancers	Preclinical	[167]
6	Epigallocatechin gallate (EGCG)	Chitosan nanoparticles	-	-	Downregulates PARP expression, increases ROS-mediated DNA damage.	Prostate and breast cancers	Preclinical	[168]
7	Genistein	Genistein–gold nanoparticles conjugates (Gen@AuNPs)	65 ± 1.7	−35 ± 2.5	Enhanced antiproliferative effect; PARP/ADPr mechanisms (e.g., cleaved PARP-1 or PARP arbitrated ADPr) yet to be investigated.	Prostate cancer	Preclinical (in vitro: PC3, DU145, and LNCaP cell lines)	[169]
8	Silibinin	No nano-conjugate reported	-	-	Prevents chemically induced lung tumors; overcomes drug resistance & metastatic traits; inhibits STAT3 in tumor and microenvironment-PARP/ADPr involvement needs further investigation.	Lung cancers	Preclinical	[170]
9	Withaferin A (WA)	Gold nanoparticles (AuNP) conjugated with dexamethasone (GR ligand) and withaferin A (Au-Dex-WA nanoconjugate).	-	-	Glucocorticoid receptor (GR)-dependent cytotoxicity, epithelial–mesenchymal transition (EMT) reversal, ATP-binding cassette sub-family G member 2 (ABCG2) downregulation; potential PARP/ADPr involvement (needs further investigation).	Mouse melanoma (EMT reversal, tumor regression); also studied in breast, lung (NSCLC), glioblastoma.	Au-Dex-WA nanoconjugate remains preclinical; WA tested in early-phase clinical studies.	[171]
10	Sulforaphane	Ultra deformable vesicles (ethosomes^®^, transfersomes^®^)	102 ± 6	−21 ± 2	ROS-mediated DNA damage and apoptosis via DR5, AP-1, MAPKs, mitochondrial dysfunction, and NF-κB inhibition; PARP/ADPr involvement yet to be explored.	Skin cancer (melanoma, SK-MEL-28)	Preclinical in vitro (Melanoma cell lines) only	[172]
11	Berberine	Poly (amidoamine) (PAMAM) dendrimer encapsulated and conjugated formulation	-	-	Induced apoptosis via mitochondrial dysfunction, ROS generation, and modulation of Bcl-2 family proteins; possible PARP cleavage during apoptotic cascade (specific role of ADPr/PARP pathway not investigated—needs further exploration.	Cervical cancer	Preclinical (in vitro—HeLa cells; in vivo—mouse xenograft model).	[173]
12	Piperine	Piperine-loaded hydroxyapatite, polymeric, and lipid nanoparticles; also, curcumin–piperine nanoparticle combinations.	63.73 ± 1.07	−20.46	Piperine triggers DNA damage and caspase-dependent PARP-1 cleavage in apoptosis; nanoparticle delivery enhances bioavailability and sustained release, though ADPr signaling needs further investigation.	Colon, prostate, and breast cancers	Preclinical models	[174,175]
13	Baicalein	Selenium–Baicalein nanoparticles (ACM-SSe-BE), coated with A549 cell membrane for homologous targeting	135.2 ± 2.52	−32.23 ± 1.19	Enhances ROS generation, promotes apoptosis and proliferation inhibition; possible ADPr/PARP involvement remains to be further investigated	A549 (non-small-cell lung cancer)	Preclinical (in vitro and in vivo in animal models)	[176]
14	Apigenin	Polymer–lipid hybrid nanoparticles (PLHNPs), macrophage-membrane-coated PEG micellar system (m@PEG-AGN), nanocrystals, micelles, liposomes, poly (lactic-co-glycolic acid) (PLGA)	125.73 ± 5.57	−26.71 ± 1.93	Causes cell cycle arrest, ROS-induced DNA damage, apoptosis; suppresses metastasis (MMP/Akt); PARP signaling through ADPr in physiological/pathophysiological context needs further investigation	Breast cancer (Triple-negative), colorectal carcinoma, and other cancer cell lines, often in vitro and/or in animal models	Apigenin remains at the preclinical stage. However, nanoformulations improve solubility, bioavailability & targeting, showing promise for clinical use	[177,178,179]

## Data Availability

Not applicable.

## Abbreviations

The following abbreviations are used in this manuscript:

TME	tumor microenvironment
CAM	complementary and alternative medicine
NSB	nano swarna bhasma
ADPr	ADP-ribosylation
ART	ADP-ribosyl transferases
NAD^+^	β-nicotinamide adenine dinucleotide
PARP	poly (ADP-ribose) polymerase
3D QSAR	three-dimensional quantitative structure–activity relationship
CDK1	cyclin-dependent kinase 1
Bax	Bcl-2-associated X protein
BRCA1	breast cancer gene 1
BRCA2	breast cancer gene 2
SIRT6	sirtuin 6
BAF170	BRG1-Associated Factor 170
IL	interleukin
NF-κB	Nuclear factor kappa-light-chain-enhancer of activated B cells
RANKL	receptor activator of NF-kappa B ligand
MDR	multidrug resistance
MAPK	mitogen-activated protein kinase
ERK	extracellular signal-regulated kinase
PKA	protein kinase A
TNF-α	tumor necrosis factor-alpha
RAD51	radiation sensitive 51.
Akt	protein kinase B
Bcl-2	B-cell lymphoma 2
COX-2	cyclooxygenase-2
MMP-9	matrix metalloproteinase-9
STAT3	signal transducer and activator of transcription 3
ROS	reactive oxygen species
EGCG	epigallocatechin-3-gallate
WFA	Withaferin A
BRAF	v-Raf murine sarcoma viral oncogene homolog B1
Raf-1	v-Raf-1 murine leukemia viral oncogene homolog 1
HSP	heat shock protein
NHEJ	non-homologous end joining
CBP	CREB-binding protein
p300	E1A-associated protein p300
HATs	histone acetyltransferases
ATR	ataxia telangiectasia and Rad3-related
DR4	death receptors 4
DR5	death receptors 5
γH2AX	phosphorylated histone H2A variant X
p65/RelA	v-Rel avian reticuloendotheliosis viral oncogene homolog A
BEL-7402	human hepatocellular carcinoma cell lines
LDH	lactate dehydrogenase
BAD	Bcl-2-associated death promoter
PEL	primary effusion lymphoma
ER	endoplasmic reticulum
JNK	c-jun N-terminal kinase
PI3K	phosphoinositide 3-kinase
mTOR	mammalian target of rapamycin
MCF-7	Michigan Cancer Foundation-7
Bcl-xL	B-cell lymphoma-extra large
CSCs	cancer stem cells
PEITC	phenethyl isothiocyanates
HER2	human epidermal growth factor receptor 2
EGFR	epidermal growth factor receptor
AP-1	activator protein 1
HIF-1α	hypoxia-inducible factor 1-alpha
iNOS	inducible nitric oxide synthase
FDA	food and drug administration
TAMs	tumor-associated macrophages
RNS	reactive nitrogen species
AGEs	advanced glycation end products
RAGE	receptor for advanced glycation end-products
NSAIDs	nonsteroidal anti-inflammatory drugs
EGCG	epigallocatechin gallate
DDR	DNA damage response
CRISPR	clustered regularly interspaced short palindromic repeats
Cas9	CRISPR-associated protein 9
ZFNs	zinc finger nucleases
TALENs	transcription activator-like effector nucleases
DSBs	double-strand breaks
